# Complete genome sequence of the bacterium *Desemzia incerta* isolated from the dorsal skin of a Yucatan pig

**DOI:** 10.1128/MRA.00519-23

**Published:** 2023-11-03

**Authors:** Monica Wei, Simon A. B. Knight, Laurice Flowers, Jasmine Walsh, Elizabeth Grice

**Affiliations:** 1 Department of Dermatology, University of Pennsylvania, Perelman School of Medicine, Philadelphia, Pennsylvania, USA; University of Maryland School of Medicine, Baltimore, Maryland, USA

**Keywords:** skin microbiota, *Desemzia incerta*, antagonism, porcine skin

## Abstract

We have shown previously that an isolate of *Desemzia incerta* from porcine skin has antimicrobial activity against methicillin-resistant *Staphylococcus aureus*. We present here the complete *D. incerta* genome containing one circular chromosome and five circular plasmids.

## ANNOUNCEMENT

*Desemzia incerta* was previously isolated from ovaries of the *Tibicen linnei* cicada (1). Here, we isolated *D. incerta* strain EGM1-39 from the dorsal skin of a female Yucatan pig housed at the Perelman School of Medicine (IACUC protocol #806346). We previously reported this isolate to exhibit antimicrobial activity against methicillin-resistant *Staphylococcus aureus* (2).

A sterile foam swab soaked in PBS was rubbed over the pig dorsum (40 cm^2^ area) and replaced into 0.3 mL PBS. This was diluted 1:10 in PBS, and 0.1 mL was plated on blood agar (BA) and incubated at room temperature for 3 days. Single colonies of unique morphology were streaked onto a second BA plate. Isolates were identified by colony PCR of the 16S ribosomal RNA region (8F and 1391R) (3). The resulting 1,229 base sequence from one such isolate was identified by NCBI Blast as *D. incerta* (Accession MH000676.1) as the top “hit” at 99.4% identity.

To prepare genomic DNA for Illumina sequencing, *D. incerta* was streaked from a glycerol stock to single colonies on BA. One colony was inoculated into 3 mL Tryptic Soy Broth (TSB) and incubated at 37°C without shaking. Genomic DNA was extracted using the ZymoResearch QuickDNA Fungal/Bacterial MicroPrep Kit. Illumina library preparation and sequencing were performed by the CHOP Microbiome Center, using the Illumina DNA Prep Kit and unique dual indexes (Illumina Nextera Index Kit) at 1:4 scale reaction volume, and sequenced on the Illumina HiSeq 2500. Trim-galore (4) v 0.2.4 was used to trim adapter sequences. Default parameters were used for all software unless otherwise specified. Quality control of reads was performed before and after read trimming using fastQC (5) v 0.11.8 settings. This workflow generated 2,080,215 paired reads of length 2 × 125 bp and 410,238,063 bp of sequence (172.2× coverage).

Genomic DNA for Oxford Nanopore Technologies (ONT) sequencing was prepared from a separate 3 mL culture, inoculated from glycerol stocks as described above, and sub-inoculated into 50 mL TSB. Genomic DNA was extracted using the NEB Monarch HMW DNA Extraction Kit for tissue using the standard input workflow for Gram-positive bacteria. For lysis, 80 uL of 100 mg/mL lysozyme in STET buffer was used, with no other modifications to manufacturer protocol. Library preparation and ONT sequencing of high-molecular weight DNA were performed by SeqCenter. Libraries were prepared using the ONT Genomic DNA by Ligation kit and sequenced on an ONT MinION with R9 flow cells (R9.4.1). Base calling was performed using Guppy (6) v 5.0.16 in high-accuracy base calling mode. PoreChop (7) v 0.2.4 was used to trim adapter sequences. This workflow generated 120,112 reads and 431,325,515 bp (181.1× coverage) of sequence with an N_50_ of 11,956 bp.

*De novo* genome assembly with both Illumina and ONT reads was performed in Unicycler v 0.4.8 (8) with default settings. This assembly contains one chromosome (2,381,418 bp) and five plasmids (13,025 bp, 7,925 bp, 7,255 bp, 5,721 bp, and 4,357 bp) which are all reported as circular by Unicycler. The total GC content is 38.0%. The chromosome and plasmids were annotated using PGAPv6.5 (9) and are predicted to contain 2,224 coding sequences, 83 tRNAs, and 22 rRNAs.

## Data Availability

Sequences are available in NCBI databases under BioProject PRJNA974032, Biosample SAMN35158552, GenBank CP126128.1 (chromosome), NZ_CP126129.1 (plasmid 1), NZ_CP126130.1 (plasmid 2), NZ_CP126131.1 (plasmid 3), NZ_CP126132.1 (plasmid 4), NZ_CP126133.1 (plasmid 5), SRA SRR24674626 (long read), and SRR24710524 (short read).
